# *Plp1* in the enteric nervous system is preferentially expressed during early postnatal development in mouse as DM20, whose expression appears reliant on an intronic enhancer

**DOI:** 10.3389/fncel.2023.1175614

**Published:** 2023-05-24

**Authors:** Pankaj Patyal, Daniel Fil, Patricia A. Wight

**Affiliations:** Department of Physiology and Cell Biology, University of Arkansas for Medical Sciences, Little Rock, AR, United States

**Keywords:** enteric nervous system (ENS), myelin proteolipid protein gene *(Plp1)*, DM20, intestine, gene expression, enhancer

## Abstract

Recently, the myelin proteolipid protein gene (*Plp1*) was shown to be expressed in the glia of the enteric nervous system (ENS) in mouse. However, beyond this, not much is known about its expression in the intestine. To address this matter, we investigated *Plp1* expression at the mRNA and protein levels in the intestine of mice at different ages (postnatal days 2, 9, 21, and 88). In this study, we show that *Plp1* expression preferentially occurs during early postnatal development, primarily as the DM20 isoform. Western blot analysis indicated that DM20 migrated according to its formula weight when isolated from the intestine. However, mobilities of both PLP and DM20 were faster than expected when procured from the brain. The 6.2hPLP(+)Z/FL transgene, which uses the first half of the human *PLP1* gene to drive expression of a *lacZ* reporter gene, recapitulated the developmental pattern observed with the native gene in the intestine, indicating that it can be used as a proxy for *Plp1* gene expression. As such, the relative levels of β-galactosidase (β-gal) activity emanating from the 6.2hPLP(+)Z/FL transgene suggest that *Plp1* expression is highest in the duodenum, and decreases successively along the segments, toward the colon. Moreover, removal of the wmN1 enhancer region from the transgene (located within *Plp1* intron 1) resulted in a dramatic reduction in both transgene mRNA levels and β-gal activity in the intestine, throughout development, suggesting that this region contains a regulatory element crucial for *Plp1* expression. This is consistent with earlier studies in both the central and peripheral nervous systems, indicating that it may be a common (if not universal) means by which *Plp1* gene expression is governed.

## Introduction

The myelin proteolipid protein gene (*Plp1*) is abundantly expressed by oligodendrocytes of the central nervous system (CNS), where it encodes the most abundant protein in CNS myelin (Jahn et al., 2009). The gene is also expressed by other cell types, generally at lower levels (reviewed in Wight and Dobretsova, 2004). Myelination in the peripheral nervous system (PNS) and CNS regulates motor, sensory, and cognitive functions (Pereira et al., 2012; Stadelmann et al., 2019) whereas there is no evidence of myelination in the ENS (Rao et al., 2015). Recently, *Plp1* expression was noted in enteric glia (Rao et al., 2015; Grundmann et al., 2019; Baghdadi et al., 2022). Indeed it, as well as the calcium-binding protein S100β, glial fibrillary acid protein (GFAP), and the transcription factor SOX10 have been used as molecular markers of enteric glial cells (Baghdadi and Kim, 2023). However, our current knowledge of *Plp1* expression in enteric glia is rather limited and primarily based on the expression of fluorescent proteins (e.g., GFP) driven by the *Plp1* promoter in transgenic mouse models (Rao et al., 2015; Belkind-Gerson et al., 2017). As well, the *Plp1* promoter has been used to drive the expression of tamoxifen-inducible Cre recombinase in enteric glia in order to ablate these cells *via* imposed expression of diphtheria toxin subunit A in Rosa26^DTA^ mice (Rao et al., 2017). Yet, our current knowledge with respect to the expression of the endogenous *Plp1* gene in the gastrointestinal tract is limited to a couple of studies, which demonstrate expression at the protein level in the ileum and colon of mice at 28 days of age (Rao et al., 2015), and in stomach and cecum of adult mice (Grundmann et al., 2019). Recent findings by Woods et al. (2023) suggest that *Plp1* is involved in the regulation of intestinal motility and barrier function in old-age mice. Beyond this, not much is known about *Plp1* expression in the intestine, including its developmental profile, the types of splice products generated, whether the level of expression varies between the different segments, and the mechanisms governing its expression in this tissue. These subjects are examined in the current study.

Far more is known about *Plp1* expression in extra-intestinal tissues, particularly the CNS. The gene is located on the X chromosome and contains seven major exons distributed across 15.6 kb in mouse (reviewed by Wight and Dobretsova, 2004). Due to alternative splicing with exon 3, two major isoforms are generated in the brain: PLP, a 276 amino acid polypeptide, and a similar protein, referred to as DM20, which lacks PLP residues 116 to 150 (Nave et al., 1987). Secondary (less abundant) transcripts are also formed in select species through the incorporation of minor exons that lie in what is typically defined as *Plp1* intron 1. Two such exons are present in the gene from a mouse (*mPlp1*) which are referred to as exon 1.1 and exon 1.2, respectively. Exon 1.1 lies within the proximal portion of *mPlp1* intron 1 (Bongarzone et al., 1999), while exon 1.2 is located toward the distal end (Li et al., 2009). Incorporation of exon 1.1 leads to the generation of additional isoforms being produced, designated as srPLP and srDM20 for soma-restricted (sr), since these proteins were confined to the cell body of mature oligodendrocytes and not targeted to the plasma membrane and compact myelin as the “classic” (PLP and DM20) products (Bongarzone et al., 1999, 2001); sr versions of the proteins contain an additional 12 residues (counting the initiator methionine) at the N-terminus. The inclusion of *mPlp1* exon 1.2 was first noted in transcripts isolated from the mouse-derived Leydig cell line, TM3 (Li et al., 2009); it is unknown whether exon 1.2-containing transcripts are translated into protein products. The current study identifies the types of transcripts produced in the mouse intestine (i.e., classic and/or splice variants).

Previously, our group demonstrated that an enhancer (wmN1) located within *Plp1* intron 1 is required for the expression of *Plp1-lacZ* constructs in the CNS and PNS of transgenic mice (Hamdan et al., 2018; Patyal et al., 2020). The 1.2-kb wmN1 region was first identified using an enhancer-trap/controlled transgenesis approach (Tuason et al., 2008). Results presented here suggest that the wmN1 enhancer region is also required for *Plp1* expression in the ENS.

## Materials and methods

### Animals

Mice used in this study were obtained from our breeding colonies of *PLP1-lacZ* transgenic mice, which use the first half of the human *PLP1* gene to drive a *lacZ* expression cassette. The founder mice were generated onsite at the Transgenic Mouse Core Facility and lines were established on the C57BL/6 genetic background as previously described (Hamdan et al., 2018). The transgenic lines were maintained in the hemizygous state in order to produce non-transgenic (wild-type, WT) littermates, which were used to investigate levels of endogenous *Plp1* gene expression. Mice were housed in a federally approved Division of Laboratory Animal Medicine facility at the University of Arkansas for Medical Sciences on ventilated cage racks in a controlled environment (temperature: 22°C; humidity: 30–50%) on a 14 h light/10 h dark cycle, with (irradiated) food and water (treated with a reverse osmosis system) provided *ad libitum*. In addition to the standard pine-chip bedding, breeder cages were supplemented with cotton nestlets. Breeding cages contained one male and two female breeders, with only one sex (typically the male) harboring the transgene.

Transgenic mice from Line 777 (Hamdan et al., 2018) that carry either a full length or recombined (internally truncated) *PLP1-lacZ* transgene were used in some experiments. The parental line contains the 6.2hPLP(+)Z/FL transgene which utilizes human *PLP1* (*hPLP1*) genomic DNA (proximal 6.2 kb of 5′-flanking DNA to the first 38 bp of exon 2) to drive expression of a *lacZ* reporter gene cassette. The related 6.2hPLPΔwmN1 subline is similar except that a portion of *hPLP1* intron 1 which overlaps the wmN1 region was removed from the transgene *via* Cre/*lox*P-mediated excision in mice. Specifically, 6.2hPLPΔwmN1 mice are missing *hPLP1* intron 1 position 3174-4660 from the transgene but retain the transgene integration site as the parental line (Hamdan et al., 2018). Transgenic mice were identified through PCR analysis of genomic DNA isolated from tail biopsies according to the methods of Truett et al. (2000), in conjunction with the *lacZ* primer pair described by Stratman et al. (2003).

Experimental groups were comprised of both male and female mice at a given postnatal day (P) of age. No blinding of experimental groups was performed in any experiment. Three mice per group were used in all experiments, without the exclusion of test subjects. A sample size calculation was not performed as three subjects per group was sufficient to detect statistical differences in a previous study (Hamdan et al., 2018). All procedures involving the use of mice were approved by the Institutional Animal Care and Use Committee at the University of Arkansas for Medical Sciences, in compliance with the Public Health Service Policy on Humane Care and Use of Laboratory Animals and the National Research Council's Guide for the Care and Use of Laboratory Animals, and adhered to ARRIVE guidelines (Kilkenny et al., 2010).

### Conventional reverse transcription-PCR (RT-PCR)

Mice were anesthetized deeply with isoflurane and euthanized by cervical dislocation except for mice at P2 of age which were laid (indirectly) on ice for 15–20 min to anesthetize them and then decapitated. Immediately afterward, the intestines (small intestine and colon) were dissected, flushed with ice-cold PBS, frozen in liquid nitrogen, and the tissue pulverized using a mortar and pestle and stored at −70°C until processing for isolation of RNA or protein (for western blot analysis). Total RNA was extracted using the RNeasy Mini kit (Qiagen, Valencia, CA) according to the manufacturer's directions. The concentration of RNA was determined using a NanoDrop 2000c Spectrophotometer (Thermo Scientific, Wilmington, DE). First-strand cDNA synthesis was performed with the iScript gDNA Clear cDNA Synthesis kit (Bio-Rad, Hercules, CA) per the supplier's instructions using 1 μg of total RNA in a final reaction volume of 20 μl. Afterward, the mixture was stored at −70°C until needed or used immediately as template DNA for PCR.

PCR was performed with 2 μl of the cDNA solution using the JumpStart REDTaq ReadyMix PCR Reaction Mix (Sigma-Aldrich, St. Louis, MO) according to the manufacturer's guidelines. *Plp* as well as *Dm20* associated products were generated using primers first described by Skoff et al. (2004), which target *Plp1* exon 2 (5′-GCTAATTGAGACCTATTTCTCC-3′) and exon 6 (5′-AGCAATAAACAGGTGGAAGGTC-3′). The reaction was run in a thermocycler for 30 cycles under the following stepwise conditions: 93°C for 1 min (denaturation), 68°C for 1 min (annealing), and 72°C for 2 min (extension).

Soma-restricted (sr) splice variants, which contain *Plp1* exon 1.1 sequence, were distinguished using the following primer pairs: Exon 1.1-F (5′-ACATGGCATTTAACTGTATTAACCCCTT-3′) and either PLP-R (5′-GGCATAGGTGATGCCCACAAACTTGTC-3′; spans junction between *Plp1* exons 3B and 4) or DM20-R (5′-GGCATAGGTGATGCCCACAAACGTTGC-3′; spans junction between *Plp1* exons 3A and 4) for amplification of *sr-Plp* and *sr-Dm20* transcripts, respectively. Alternatively, Exon 1.2-F (5′-TCCACAGGAAACCTAGACGAACCCAA-3′) was used as a sense primer, and either PLP-R or DM20-R as the antisense primer to discern any splice variants that incorporate *Plp1* exon 1.2. The PCR conditions to detect any of these *Plp1* splice variants were as follows: a single step of 94°C for 4 min; 40 repetitive cycles of 94°C for 75 s (denaturation), 60°C for 75 s (annealing), and 72°C for 90 s (extension); and a final step of 72°C for 7 min.

PCR products were fractionated on a 1% agarose gel in TAE buffer (40 mM Tris pH 8.0, 20 mM Acetate, and 1 mM EDTA). Images of gels were captured on a ChemiDoc MP Imaging System (Bio-Rad).

### Reverse transcription-quantitative PCR (RT-qPCR) analysis

First-strand cDNA synthesis was performed as described in the preceding section except that only 0.98 μg of total RNA was used in the reaction. Once complete, 180 μl of nuclease-free water (Life Technologies, Carlsbad, CA) was added to dilute the cDNA mixture 10-fold, and the solution was stored at −20°C until further use. Quantitative PCR (qPCR) was performed in a StepOnePlus Real-Time PCR System (Applied Biosystems, Foster City, CA) using 2 μl of dilute cDNA mixture with the TaqMan Fast Advanced Master Mix (Applied Biosystems) and a particular TaqMan Gene Expression Assay (Applied Biosystems) for a final volume of 10 μl. The supplier's recommended reaction conditions were used which consisted of a hot start at 95°C for 20 s followed by 40 reiterative cycles of 95°C for 1 s and 60°C for 20 s. Custom primer/probe sets (TaqMan Gene Expression Assays) for *Plp1*-related transcripts (see Supplementary Figure 1 for more details) were as follows: *Plp* and *Dm20*, combined (PLP/DM20-F sense primer: 5′-CAAGACCTCTGCCAGTATAGG-3′; PLP/DM20-R antisense primer: 5′-CAGCAATAAACAGGTGGAAGG-3′; probe: 5′-TGCCAGAATGTATGGTGTTCTCCCAT-3′); *Plp*-specific (PLP-F sense primer: 5′-CTAGGACATCCCGACAAGTTT-3′; PLP-R antisense primer: 5′-GGTGGTCCAGGTATTGAAGTAA-3′; probe: 5′-TTGTATGGCTCCTGGTGTTTGCCT-3′); *Dm20*-specific (DM20-F sense primer: 5′-CCTGAGCGCAACGTTTGT-3′; DM20-R antisense primer: 5′-GGTGGTCCAGGTATTGAAGTAA-3′; probe: 5′-TTGTATGGCTCCTGGTGTTTGCCT-3′). The custom primer/probe set for the detection of transcripts encoded by the *hPLP1-lacZ* transgene was as follows: (*hPLP1*) Exon 1-F sense primer, 5′-CTGAACAAAGTCAGCCACAAAG-3′; LacZ-R antisense primer, 5′-GTTGAAACGCTGGGCAATATC-3′; probe, 5′-ACATGGGCTTGT-3′. Primer probe/sets for the housekeeping genes, *18S* (Mm03928990_g1) and *Gusb* (Mm01197698_m1), were commercially available from Applied Biosystems (Cat# 4448489 and 4331182, respectively). PCR reactions were run in duplicate per cDNA sample for every primer/probe set. The relative level of gene expression was calculated using the 2^−Δ*ΔCt*^ method. Results are presented as the mean level ± SD relative to that from the *18S* reference gene for biological triplicates. Statistical analysis between levels of *Plp* and/or *Dm20* mRNA expression at various ages was determined by one-way ANOVA with Tukey's procedure using the GraphPad Prism 9.3.1 software (Dotmatics, San Diego, CA). Alternatively, statistical analysis between levels of *lacZ* mRNA expressed by the parental 6.2hPLP(+)Z/FL and rearranged 6.2hPLPLΔwmN1 transgene at a given age was determined with a two-tailed Student's *t*-test (unpaired). The threshold to attain significance was set at 0.05 (*p*-value).

### Western blot analysis

Pulverized intestinal tissue containing the small intestine and the colon (see earlier section for more details) was homogenized in 4% SDS (250 mg tissue/5 ml), centrifuged at 12,000 *g* for 10 min at 4°C, and the supernatant was then transferred to a fresh tube. Lysate protein concentrations were determined using the Pierce BCA Protein Assay Kit (ThermoFisher Scientific/Pierce Biotechnology, Rockport, IL). Proteins were denatured in gel loading buffer (31.25 mM Tris pH 6.8, 2% SDS, 5% glycerol, 0.05 mg/mL bromophenol blue, 0.785% β-mercaptoethanol) for 10 min at 55°C, then subjected to electrophoresis on a 4–15% Mini-Protean TGX Gel (Bio-Rad), and, finally, transferred to a nitrocellulose membrane (0.45 μm pore size). Membranes were blocked with 5% non-fat dry milk in Tris-buffered saline with Tween-20 (TBST; 20 mM Tris pH 7.6, 150 mM NaCl, 0.1% Tween-20) for 1 h at room temperature, washed three times with TBST (5 min each), and incubated overnight at 4°C with primary antibody (rat anti-PLP/DM20 monoclonal antibody was provided by Dr. Wendy Macklin) diluted 1:5000 in the blocking solution. Blots were washed three times with TBST at room temperature (5 min/wash) and then incubated with a secondary antibody (Invitrogen, Waltham, MA, Cat# 31470) diluted 1:10,000 in blocking solution. After imaging, the membrane was washed in TBST at room temperature for 5 min and then incubated overnight at 4°C with mouse anti-β-actin antibody (Invitrogen, Cat# MA5-15739) diluted 1:10,000 in blocking solution. The next day the membrane was washed three times in TBST (5 min each) and incubated for 1 h at room temperature with a secondary antibody (Invitrogen, Cat# G21040) diluted 1:10,000 in blocking solution. Immunoreactive bands were visualized with the SuperSignal West Dura Luminol/Enhancer Solution (ThermoFisher Scientific/Pierce Biotechnology, Cat# 1856145). Images were captured on a ChemiDoc MP Imaging System and analyzed with Image Lab 6.0.1 Software (Bio-Rad). Statistical analysis between the relative amounts of DM20 at various ages was determined by one-way ANOVA with Tukey's procedure using the GraphPad Prism 9.3.1 software. The threshold to attain significance was set at 0.05 (*p*-value).

### β-galactosidase enzyme assay

Mice were anesthetized *via* inhalation of isoflurane and then euthanized by cervical dislocation. Intestines were rapidly dissected, flushed clean with ice-cold PBS, and homogenized in Lysis Solution [0.2% Triton X-100 and freshly added phenylmethylsulfonyl fluoride (0.2 mM) and leupeptin (5 μg/ml) in 100 mM potassium phosphate, pH 7.8]. In some cases, the small intestine and colon were homogenized together, whereas in other cases the individual segments of the small intestine (duodenum, jejunum, and ileum) and colon were homogenized separately. The homogenate was centrifuged at 12,500 g for 10 min at 4°C and the resulting supernatant was incubated at 48°C for 1 h to inactivate any endogenous β-galactosidase (β-gal) activity according to the methods of Young et al. (1993). The lysate was then centrifuged at 12,500 *g* for 5 min at 4°C and the supernatant (10 μl) was assayed for β-gal activity in triplicate using the Galacto-Light Plus Kit as previously described (Pereira et al., 2013). The protein concentration of the lysates was determined as described in the methods for western blot analysis. Results are presented as the mean ± SD of β-gal activity (relative light units; RLU) per μg of total protein for three transgenic mice of a given genotype/age, minus the background. The mean background level (RLU/μg protein) was obtained from three non-transgenic (wild-type) littermates. Statistical analysis between the levels of β-gal activity produced by 6.2hPLP(+)Z/FL and 6.2hPLPLΔwmN1 in duodenum at given age was determined by a two-tailed Student's *t*-test (unpaired) using the GraphPad Prism 9.3.1 software. Statistical analysis between levels of β-gal activity from 6.2hPLP(+)Z/FL in the various segments of the intestine was determined by one-way ANOVA with Tukey's procedure. The threshold to attain significance was set at 0.05 (*p*-value).

## Results

### The peak of *Plp1* mRNA expression in the intestine occurs during the early postnatal period of mouse development, primarily as the *Dm20* splice variant

**Figure 1:**
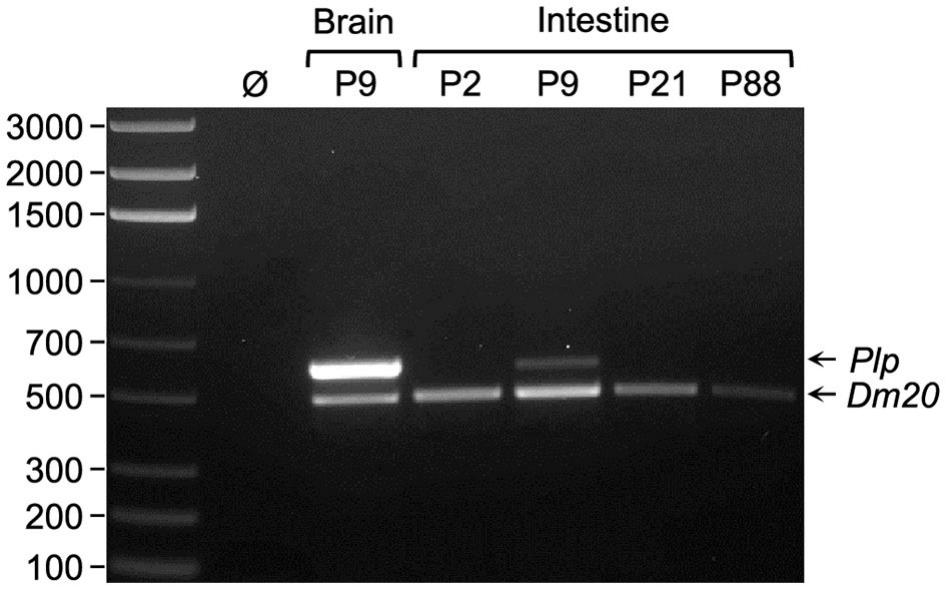
*Dm20* splice variant is the major transcript expressed by *Plp1* in intestine. RT-PCR analysis was performed using RNA isolated from either brain or intestine of mice at the indicated postnatal days of age. The primer pair utilized amplifies sequences between *Plp1* exons 2 and 6, thus it is possible to distinguish the *Plp* amplicon (600 bp) from that of *Dm20* (495 bp). An image of a representative gel containing the RT-PCR product(s) generated from the indicated tissues of a given mouse (a total of three mice were analyzed) is shown. The results demonstrate that the *Dm20* splice variant is the major transcript expressed by *Plp1* in the intestine at all ages examined (highest level observed at P9), whereas *Plp* mRNA predominates over the *Dm20* transcript in the brain at P9. Ø indicates water in lieu of template (cDNA) in the PCR reaction. The left lane contains a ladder of DNA size markers (bp).

RT-PCR analysis was performed with RNA isolated from mice at several ages (P2, P9, P21, and P88) to assess whether the expression of transcripts encoded by *Plp1* is regulated developmentally in the intestine. For the purposes of this study, the intestine denotes the small intestine and colon, unless specified, otherwise. As shown in Figure 1, expression was greater during the early postnatal period (P2 and P9), with mice at P9 showing the highest level of the ages tested. Expression decreased at P21 and was virtually undetectable at P88. *Dm20* mRNA was the major splice species formed in the intestine (although a small amount of *Plp* mRNA was detected at P9), unlike in the brain where the *Plp* transcript predominates at P9. Because the primers used in the RT-PCR analysis were directed at sequences in *Plp1* exons 2 and 6, the contribution (if any) of splice variants that possess supplementary exons within *Plp1* intron 1 DNA could not be ascertained. Therefore, additional RT-PCR analysis was performed, which demonstrates that these supplementary exons are not utilized to generate ancillary splice variants in the intestine, unlike in the brain (Supplementary Figures 2, 3).

**Figure 2:**
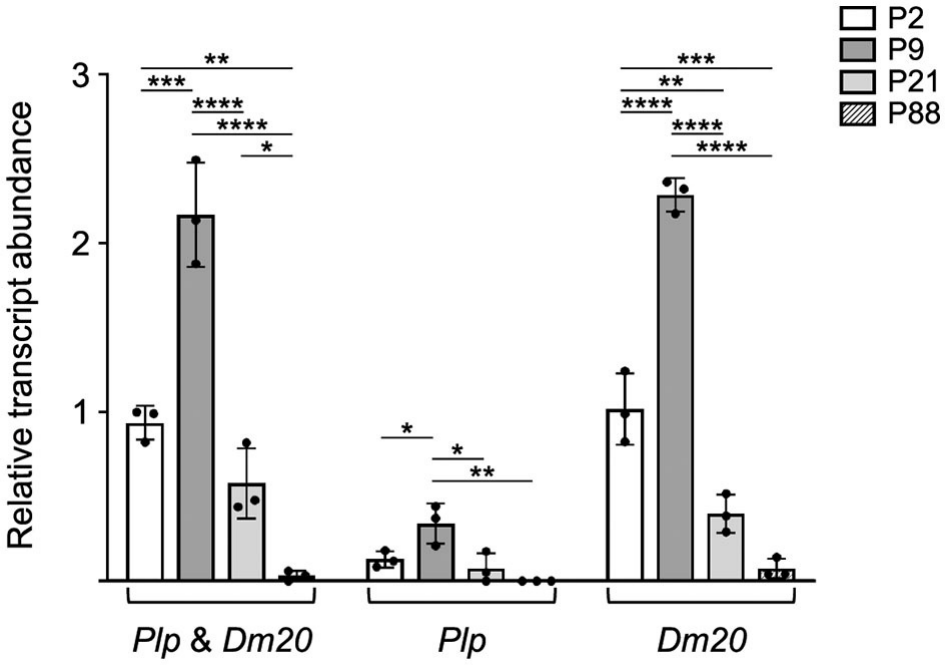
*Plp1* expression in the intestine is higher during the early postnatal period in mouse than at later stages. RT-qPCR analysis was employed to quantitatively assess the relative levels of *Plp1* expression in the intestine from mice at the indicated postnatal days of age. Custom designed assays were used in order to measure the levels of *Plp* and *Dm20* mRNA, separately, or in combination (*Plp* and *Dm20*). The results demonstrate that the levels of *Plp* and *Dm20* mRNA were more prominent during the early postnatal period of development (highest at P9) and declined at the later ages examined, with the *Dm20* splice variant accounting for the vast majority of *Plp1* expression. Results are reported as the fold mean ± SD of *Plp1* expression (*n* = 3 mice per age) relative to that from the *18S* reference gene, with the combined amount of *Plp* and *Dm20* mRNA at P2 arbitrarily set at 1-fold. Significant difference (*p ≤ 0.05; **p ≤ 0.01; ***p ≤ 0.001; ****p ≤ 0.0001) between mRNA levels at various ages by one-way ANOVA with Tukey's procedure.

To better quantitate the levels of *Plp1*-derived transcripts during development, RT-qPCR analysis was performed. Custom-designed assays were used in these studies to determine the level of *Plp* and *Dm20* transcripts combined, as well as independently. As shown in Figure 2, the level of *Plp1* expression in the intestine was higher during the early postnatal period, with a greater amount at P9 than at P2. However, by P21 the level decreased and was low at P88. Similar to the results in Figure 1, *Dm20* was the major mRNA species generated at all ages tested (Figure 2).

### DM20 from the intestine migrates as expected by SDS-PAGE, but *Plp1* products from the brain migrate anomalously

**Figure 3:**
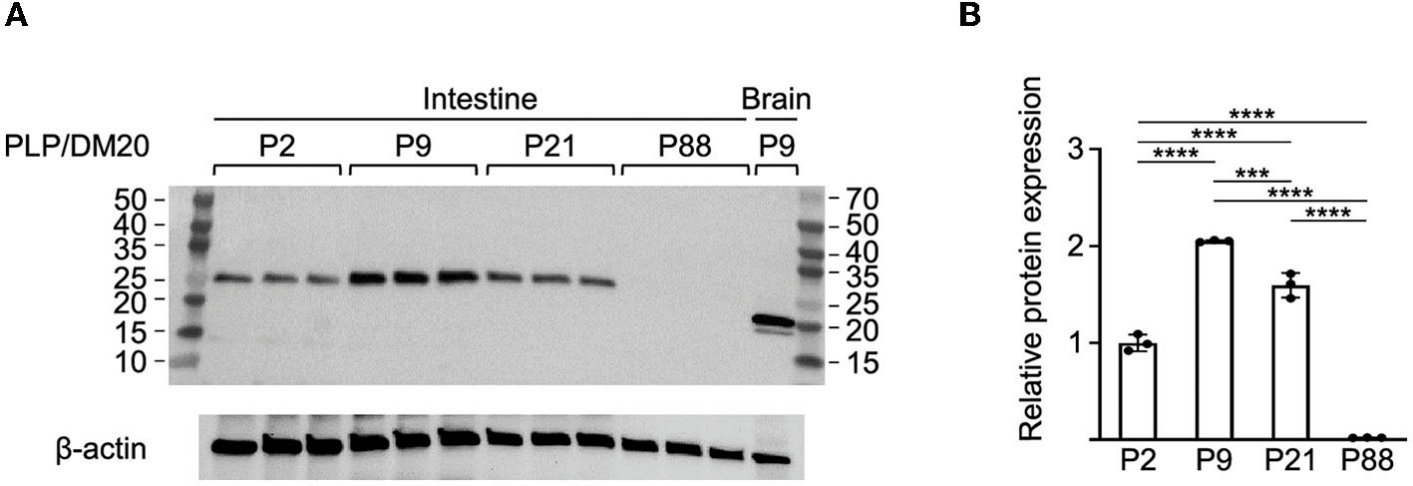
Western blot analysis of *Plp1* expression in the intestine during postnatal development. **(A)** Protein was isolated from the intestine and the brain of mice at the indicated ages and the relative levels of PLP and DM20 protein were assessed by western blot analysis using an antibody that recognizes both isoforms. In total, 10 micrograms of total protein were loaded in the lanes containing protein isolated from the intestine, while only 5 μg were used in the lane containing protein from the brain. The blot was incubated first with an anti-PLP/DM20 antibody and subsequently with an anti-β-actin antibody. The immunoblot indicates the highest level of *Plp1* expression occurs at P9 of the ages examined, and migrates in accordance with the formula weight for DM20. The mass (kDa) of protein markers (outer lanes) is indicated. **(B)** Quantification of the western blot results in **(A)**, for the intestine. Plotted is the mean ratio ± SD of DM20 to β-actin (*n* = 3) at a given age, with the amount at P2 arbitrarily set at 1-fold. Significant difference (***p ≤ 0.001; ****p ≤ 0.0001) between the amount of DM20 at various ages by one-way ANOVA with Tukey's procedure.

Western blot analysis was performed to assess whether the developmental expression of *Plp1* encoded proteins in the intestine roughly mirrored the pattern observed for mRNA. As shown in Figure 3, the level of protein was highest at P9, with lower amounts detected at P21 followed by P2. An even lower amount of the protein was present at P88 as demonstrated by “overexposing” the blot (Supplementary Figure 4). Unlike the homogenate procured from the brain, only a single immunoreactive band was observed with homogenates prepared from the intestine. These results, together with those in Figure 1, suggest that DM20 is the primary isoform present in the intestine. Curiously, the mobility of immunoreactive bands differed between the intestine and the brain, with faster mobilities observed for both PLP and DM20 when isolated from brain than DM20 isolated from the intestine. In fact, brain-derived DM20 was named in part for its apparent molecular weight (~20 kDa) by SDS-polyacrylamide gel electrophoresis (Agrawal et al., 1972). However, its actual formula weight is larger (~26 kDa), which is consistent with the size of the immunoreactive band observed with extracts prepared from the intestine (Figure 3A). Previous studies have shown that *Plp1* products in the brain are covalently linked to long-chain fatty acids *via* several cysteine residues (Stoffyn and Folch-Pi, 1971; Bizzozero and Good, 1990; Weimbs and Stoffel, 1992). However, differences in the acylation status between the tissues cannot explain the “gel shifting” observed when the proteins are isolated from the brain since a previous study demonstrated that removal of fatty acid from PLP or DM20 does not alter their electrophoretic mobility (Bizzozero et al., 1985). Other studies have revealed that it is common for membrane proteins to migrate anomalously on SDS-PAGE (Rath et al., 2009; Rath and Deber, 2013). Thus, it appears that DM20 in the intestine does not associate with the plasma membrane in a manner similar to that in the brain, if at all.

To show where within the small intestine DM20 is expressed, immunofluorescence staining was performed using transverse sections of the duodenum from a mouse at P21 of age. Enteric glial cells will have formed fully functional networks by this age (Le Berre-Scoul et al., 2017). As shown in Supplementary Figure 5, there was significant immunostaining within the submucosal and myenteric plexuses and villus with the PLP/DM20 antibody, consistent with the results of Rao et al. (2015). As expected, some of the DM20+ cells within these regions also appear to express GFAP, which is better observed with immunostaining of a whole mount section of the submucosal plexus (Supplementary Figure 5). However, the overlap is incomplete, with some cells expressing primarily one or the other of these two proteins, consistent with earlier findings by Rao et al. (2015) and the single-cell RNA sequencing (scRNA-seq) analysis by Baghdadi et al. (2022), which uncovered heterogeneity (*Gfap*^*High*^*/Plp1*^*Low*^, *Gfap*^*Low*^*/Plp1*^*High*^, and *Gfap*^*Mid*^*/Plp1*^*Low*^) between populations of enteric glial cells.

### The wmN1 enhancer region of *Plp1* intron 1 is required for the expression of a *PLP1-lacZ* transgene in the intestine

**Figure 4:**
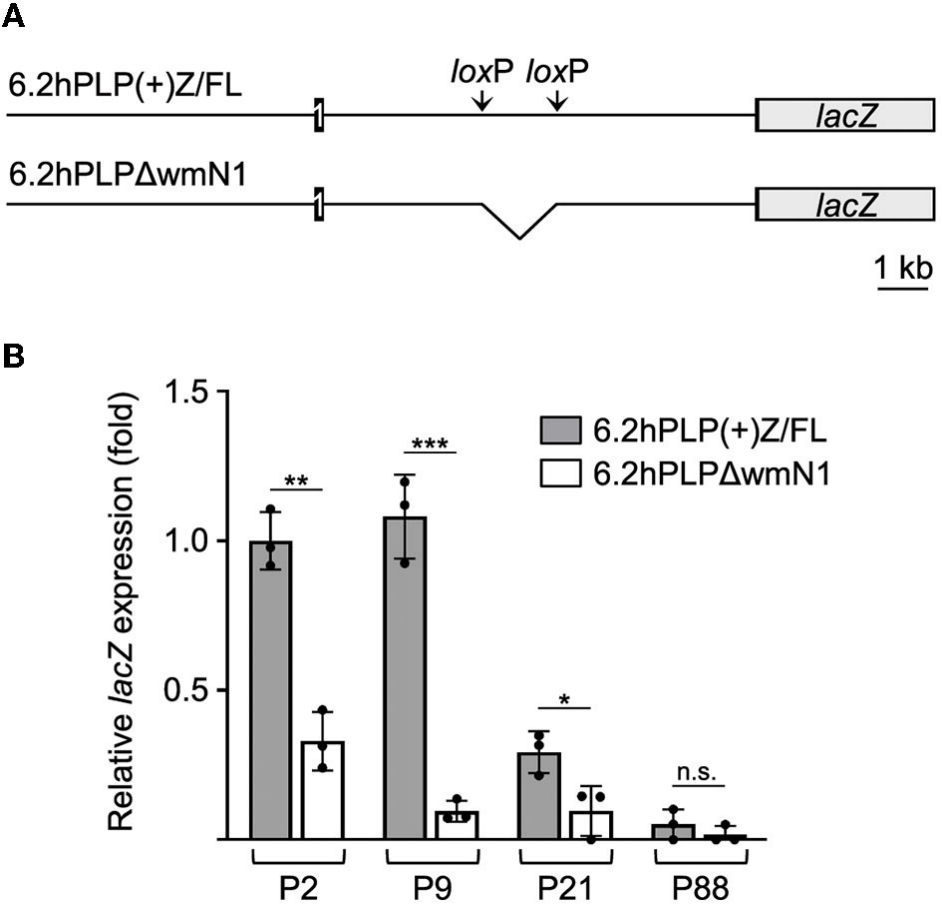
wmN1 enhancer region in *Plp1* intron 1 is required to achieve substantial levels of mRNA expression by a *PLP1-lacZ* transgene in the intestine. **(A)** Schema of the 6.2hPLP(+)Z/FL transgene which utilizes human *PLP1* (*hPLP1*) DNA (proximal 6.2 kb of 5′-flanking DNA to first 38 bp of exon 2) to drive expression of a *lacZ* reporter cassette. Black boxes denote *hPLP1* exon 1 and the beginning of exon 2. Black lines represent *hPLP1* 5′-flanking DNA and intron 1. The 6.2hPLP(+)Z/FL transgene contains added pairs of *lox*P and *Frt* (not shown) sites in *hPLP1* intron 1. The *lox*P sites border sequences orthologous to the mouse wmN1 enhancer region plus an additional 330 bp directly upstream. The rearranged 6.2hPLPLΔwmN1 subline was established through *Cre* recombinase-mediated excision of the “floxed” region from mice bearing the original (parental) transgene. **(B)** RT-qPCR analysis of transgene expression in the intestine (duodenum to the colon) from 6.2hPLP(+)Z/FL or 6.2hPLPΔwmN1 mice at the indicated ages. Loss of the wmN1 region from the transgene resulted in a significant decrease in expression at all ages examined except at P88, in which the amount produced by 6.2hPLP(+)Z/FL was low to begin with. Results are reported as the mean ± SD of *lacZ* expression (*n* = 3) relative to that from the *18S* reference gene, with the amount produced by the 6.2hPLP(+)Z/FL transgene at P2 arbitrarily set at 1-fold. Significant difference (*p ≤ 0.05; **p ≤ 0.01; ***p ≤ 0.001) between the levels of mRNA expressed by the parental 6.2hPLP(+)Z/FL transgene and the rearranged 6.2hPLPLΔwmN1 transgene, at a given age, using a two-tailed Student's *t*-test (unpaired).

**Figure 5:**
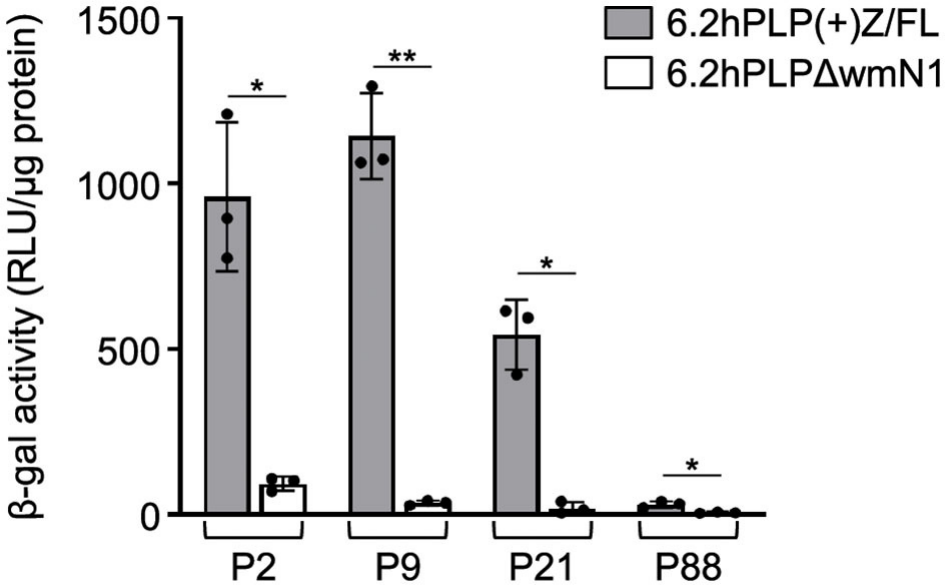
Loss of the wmN1 enhancer region from a *PLP1-lacZ* transgene results in decreased levels of β-gal activity in the intestine. The level of β-gal activity was determined in homogenates prepared from the intestine of 6.2hPLP(+)Z/FL and 6.2hPLPΔwmN1 mice at the indicated ages. Results are presented as the mean RLU/μg protein ± SD (*n* = 3) minus background (determined from corresponding wild-type littermates) per genotype/age. Removal of the wmN1 enhancer region from the transgene caused a drop in β-gal activity at all ages examined. Significance difference (*p ≤ 0.05; **p ≤ 0.01) between the levels of β-gal activity produced by 6.2hPLP(+)Z/FL and 6.2hPLPΔwmN1 for a given age, using a two-tailed Student's *t*-test (unpaired).

**Figure 6:**
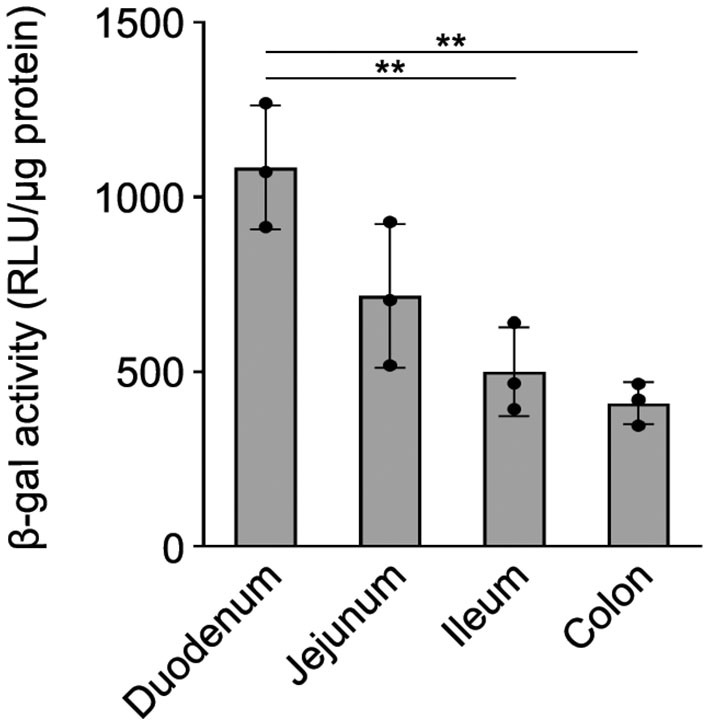
β-gal activity from 6.2hPLP(+)Z/FL varies in the segments of the intestine. The level of β-gal activity was determined in homogenates prepared from the indicated segments of the intestine with 6.2hPLP(+)Z/FL mice at P9 of age. Results are presented as the mean RLU/μg protein ± SD (*n* = 3) minus background (determined from wild-type littermates). The level of β-gal activity expression was highest in the duodenum and sequentially declined in the segments of the intestine toward the colon. Significance difference (**p ≤ 0.01) between the levels of β-gal activity in various segments of the intestine by one-way ANOVA with Tukey's procedure.

Previously, we have shown that the wmN1 enhancer region in *Plp1* intron 1 DNA is required for the expression of *Plp1-lacZ* transgenes in the CNS and PNS of mice (Hamdan et al., 2018; Patyal et al., 2020). To test whether this region is also required for expression in the intestine, two related transgenic mouse lines were examined. The parental line contains the 6.2hPLP(+)Z/FL transgene, which utilizes the first half of the human *PLP1* gene (*hPLP1*) including the promoter and all of the intron 1 sequence, to drive expression of a *lacZ* reporter cassette (Figure 4A). The 6.2hPLPΔwmN1 subline is similar except that it is missing a 1.5-kb portion of *hPLP1* intron 1, including the wmN1 region, which was deleted *in vivo* using Cre/*lox*P technology (Line 777 in Hamdan et al., 2018). RT-qPCR analysis of 6.2hPLP(+)Z/FL expression in the intestine was higher during the early postnatal period (see P2 and P9), which decreased by P21, and even further at P88 (Figure 4). Thus, the developmental pattern of the 6.2hPLP(+)Z/FL transgene recapitulates the expression profile determined for the endogenous (*Plp1*) gene (Figure 2). Loss of the wmN1 region (6.2hPLPΔwmN1) greatly diminished transgene mRNA levels at all ages tested except at P88, where the amount produced by 6.2hPLP(+)Z/FL was low to begin with (Figure 4B). Similar trends were also observed by measuring the enzymatic activity emanating from the transgene in lysates prepared from the intestine. The level of β-gal activity from the 6.2hPLP(+)Z/FL transgene was higher at the earlier postnatal ages (P2 and P9), while loss of the wmN1 region caused β-gal activity to be low at all ages examined (Figure 5). To determine if the level of *Plp1* expression varies along the intestinal tract, β-gal assays were performed using lysates prepared from different portions of the intestine from 6.2hPLP(+)Z/FL mice at P9 of age. As shown in Figure 6, the level of transgene activity was highest in the duodenum and progressively decreased along the intestinal tract toward the colon.

## Discussion

Far more is known about *Plp1* expression in the CNS than in the ENS. To address this gap, we chose to study the expression of the native gene, as well as *PLP1-lacZ* transgenes, in the intestine of mice at several postnatal ages (P2, P9, P21, and P88). RT-PCR analyses demonstrate that the alternative splice transcript, *Dm20*, accounts for most of *Plp1* expression in the intestine at these ages (Figures 1, 2). This is consistent with the work of Skoff et al. (2004), which found *Dm20* transcripts to be more abundant than *Plp* transcripts in the developing intestine of mice at embryonic day 14.5. Moreover, the *Dm20* transcript that is formed is classic—it does not contain sequence from either mouse *Plp1* supplementary exons 1.1 or 1.2, within what typically is defined as intron 1 (Supplementary Figures 2, 3). Correspondingly, we were unable to detect splice variants that incorporate sequences from a pair of unrelated supplementary exons within human *PLP1* intron 1 (exons AB and C; Sarret et al., 2010) in the intestine of 6.2hPLP(+)Z/FL transgenic mice (data not shown), although low levels of these variants are present in the brain (Patyal et al., 2023). Thus, the *Plp1* transcripts generated in the intestine are of the standard (classic) type, with *Dm20* being the major species.

Developmentally, the level of mRNA generated from the *Plp1* gene in the intestine was greater during the early postnatal period, with mice at P9 demonstrating the highest level of the ages tested (Figure 2). A similar profile was observed with the 6.2hPLP(+)Z/FL transgene (Figure 4), indicating that the transgene contains (*Plp1*) transcription element(s) sufficient to mediate temporal regulation in the intestine. By P21 of age, the amount of mRNA produced from the native gene and 6.2hPLP(+)Z/FL transgene declined significantly, and was low by P88 of age, suggesting that *Plp1* product(s) likely serve a purpose during the early postnatal period in the intestine. The developmental pattern of protein expression by *Plp1* exhibited a corresponding profile with the highest amount attained at P9, of the ages examined (Figure 3). However, the level of DM20 in the intestine was greater at P21 than at P2, which may reflect a delay between transcription and translation, or a higher degree of stability at the protein level than mRNA level. Even so, the amount of DM20 in the intestine declined between P9 and P21 of age indicating that the need for the protein lessens after the early postnatal period of development in mouse; part of the decrease may simply reflect compositional changes due to the growth of the intestinal tissue during this period (Parathan et al., 2020). Interestingly, expression of S100β and GFAP in the rat large intestine increases during the first several weeks after birth that is sustained out to at least the fifth week, postnatal, whereas expression of Sox10, another marker of enteric glia, decreases after the first week, postnatal (Cossais et al., 2016).

We could detect immunostaining for DM20 within the submucosal and myenteric plexuses and villus in the duodenum of a mouse at P21 of age (Supplementary Figure 5), similar to the finding of Rao et al. (2015). Exactly how the protein functions in the intestine is currently unknown. The ENS is not myelinated (Gershon and Rothman, 1991; Rao et al., 2015), thus DM20 in the intestine must fulfill a function unrelated to myelin. In the CNS, DM20 has been proposed to have additional roles besides being a structural component of myelin, since it is expressed embryonically, before the formation of myelin (Ikenaka et al., 1992). Based on the temporal pattern of *Plp1* expression observed in the current report with postnatal mice, and the RT-PCR results by Skoff et al. (2004) demonstrating *Plp1* expression in the developing (mouse) intestine at embryonic day 14.5 (E14.5), it is tempting to speculate that DM20 may play a role in the maturation and/or function of the developing ENS; enteric glial cells begin to form at E12 in rodents and continue to mature postnatally up to 4 weeks after birth (for a recent review see Pawolski and Schmidt, 2020). However, Woods et al. (2023) did not find any differences in ENS glial and neuronal numbers, or in glial arborization, in young (~3 months) and old (>1 year) *Plp1* null mice. Moreover, lack of *Plp1* expression in the old (but not young) mice elicited changes in gut motility and barrier function that the author's posit is being mediated through the Erk1/2 pathway (Woods et al., 2023). Enteric glial cells have multiple roles in intestinal homeostasis and regeneration (for recent reviews see Boesmans et al., 2022; Baghdadi and Kim, 2023; Sharkey and Mawe, 2023). Thus, it is possible that some of these functions may have been compromised in the old *Plp1* null mice. While we did detect a slight amount of DM20 expression in the intestine at P88 in our study, it is possible that the level may vary depending on the environment. Our mice were housed in an extremely clean facility reserved for genetically modified mice; therefore, their immune system was minimally challenged. Additionally, differences between the gut microbiota may vary between animal facilities, and thus impact DM20 expression. Kabouridis et al. (2015) showed that indigenous gut microbiota can regulate the homeostasis of enteric glial cells, while Vicentini et al. (2021) determined that the gut microbiota is essential for the maintenance of ENS integrity and affects enteric neuronal and glial cell survival.

The ENS is highly innervated by extrinsic nerve fibers. Schwann cell precursors invade the gut alongside extrinsic nerves and give rise to Schwann cells, endoneurial fibroblasts (Joseph et al., 2004), and enteric neurons (Uesaka et al., 2015). Whether DM20 is expressed in the extrinsic Schwann cells is unknown currently. However, it would not be surprising if DM20 was expressed in Schwann cells since the protein has been shown to be expressed in an array of other tissues (Nadon et al., 1997).

Our results demonstrate that DM20 migrates according to its formula weight by western blot analysis with protein extracts prepared from the intestine, unlike that from the brain; both PLP and DM20 migrated faster than expected when isolated from the brain (Figure 3). Anomalous migration with SDS-PAGE is commonly observed for membrane proteins and has been attributed to alterations in detergent binding (Rath et al., 2009). Typically, membrane proteins with multiple helical transmembrane domains migrate faster than expected, while those possessing only a single domain tend to migrate slower (Rath and Deber, 2013); PLP and DM20 possess four transmembrane domains (Popot et al., 1991; Weimbs and Stoffel, 1992). A difference in the acylation status between the proteins in the brain and intestine cannot explain the “gel shifting” observed with the brain-derived products (Figure 3), since a previous study (Bizzozero et al., 1985) established that the removal of fatty acid from PLP and DM20 does not alter their electrophoretic mobility. Perhaps the anomalous migration of the proteins obtained from the brain stems from their prior association with myelin. Whatever the underlying cause may be in the brain, it is important to note that DM20 migrates as expected when isolated from the intestine. To the best of our knowledge, only one other group (Baghdadi et al., 2022) has examined PLP/DM20 expression in the gut by western blot analysis. Our results suggest that the ~25 kDa band labeled PLP1 in the study by Baghdadi et al. is instead, DM20. Nonetheless, it is clear that in both the brain and intestine, the wmN1 enhancer region is required for the expression of a *PLP1-lacZ* transgene (Figures 4, 5; Hamdan et al., 2018). These results suggest that at least some mechanisms regulating *Plp1* transcription are preserved across a diverse assortment of cell types. Where within this region enhancer activity is emanating from remains to be determined as the sequence is greater than 1 kb. Once the sequence is better refined, candidate transcription factors can be evaluated for their possible role in mediating enhancer activity. It is interesting to note that the wmN1 region contains multiple target sites for Sox-related proteins, among a multitude of possible regulatory elements. A recent study by Kim et al. (2021) identified two distal enhancers that are important for activating *Plp1* expression in oligodendrocytes. These enhancers were not included in the 6.2hPLP(+)Z/FL transgene. In fact, these enhancers are located closer to other genes on the X chromosome. ChIP-seq studies show that these newly identified enhancer regions (termed Plp1-E1 and Plp1-E2) are bound by Sox10, Tcf7l2/Tcf4, Olig2, and Myrf (Kim et al., 2021). Interestingly, some of these target sites are also present within the wmN1 enhancer region. Thus, it is possible that some of these elements may be important for wmN1 enhancer activity, as well. Sox10 is an important factor in the development of the enteric nervous system (for review see Bondurand and Sham, 2013). Sox 10 is expressed in enteric glial cells (Young et al., 2003), and its expression is consistent with what we found for DM20, as discussed earlier. Tcf-4 (encoded by the *Tcf7l2* gene) is important for intestinal homeostasis (Korinek et al., 1998; van Es et al., 2012; Wenzel et al., 2020). Myrf is also expressed in the postnatal intestine (Baldarelli et al., 2021). Whether Tcf4 or Myrf is present in enteric glial cells remains to be determined.

In summary, the results presented in this study demonstrate that expression of the *Plp1* gene in the intestine is temporally regulated, with higher amounts attained during early postnatal development in mouse. Of the ages examined, expression was highest in the intestine of mice at P9, with comparatively higher levels observed in the duodenum (Figure 6), which progressively decreased along the different segments of the intestinal tract to the colon. Since the majority of our analyses were performed using a mixture of the small and large intestine (colon), future studies should focus on the developmental expression of *Plp1* expression in the various segments, separately, as the ENS and its components have been shown to exhibit complex heterogeneity along the intestinal wall (Boesmans et al., 2015; Nestor-Kalinoski et al., 2022; Seguella et al., 2022). While beyond the scope of this study, a complete developmental assessment of PLP/DM20 immunostaining should be performed as well; expression of the *PLP1-lacZ* transgene cannot be used as a proxy since there are not any good antibodies commercially available for bacterial β-galactosidase, and there is significant background staining in the gut when X-gal is used as a chromogenic substrate. Similar to our previous results in the CNS and PNS (Hamdan et al., 2018), the wmN1 enhancer region in *Plp1* intron 1 is required for the expression of a *PLP1-lacZ* transgene in the ENS, suggesting that the mechanisms regulating *Plp1* transcription may be conserved across cell types. DM20 is the predominant isoform expressed in the intestine. What function the protein serves in the intestine has yet to be determined, but the timing of its expression is consistent with the development of neurons and glia in the ENS, suggesting that it could possibly have a role in the proliferation and/or maturation of these cell types. The use of mice that have elevated expression (or lack thereof) of *Plp1* may be helpful in future studies to determine a role for the protein in the ENS. It is conceivable that patients with altered *Plp1* gene dosage may experience problems in the gut, in addition to the well-documented deficits noted in the CNS (Wolf et al., 1999).

## Data availability statement

The original contributions presented in the study are included in the article/Supplementary material, further inquiries can be directed to the corresponding author.

## Ethics statement

The animal study was reviewed and approved by Institutional Animal Care and Use Committee at the University of Arkansas for Medical Sciences.

## Author contributions

PP and PW conceived the study. PP and DF performed the experiments. PP and PW wrote the manuscript, with journalistic input from DF. All authors read and approved the final manuscript.
