# Supplementary material for: Plp1 in the enteric nervous system is preferentially expressed during early postnatal development in mouse as DM20, whose expression appears reliant on an intronic enhancer
Source: Front Cell Neurosci. 2023 May 24;17:1175614. doi: 10.3389/fncel.2023.1175614 (PMC10244531; doi:10.3389/fncel.2023.1175614)
Supplement: Supplementary file 1 [file Data_Sheet_1.PDF]

Immunohistochemistry

Mice at P21 of age were anesthetized with isoflurane and perfused intracardially with ice-cold Phosphate Buffered Saline (PBS) pH 7.3, followed by 200 ml of fixative (4% paraformaldehyde in PBS). The tissue that lies between the stomach and rectum was removed as a single piece and placed in PBS in a petri dish on ice. The mesenteric vascular was then gently tweezed from the GI tract through the use of forceps. The cecum was discarded and the two remaining pieces (i.e., small intestine and colon) were flushed with ice-cold PBS, until clean. The small intestine was divided into thirds along its length from the proximal end near the stomach into segments that correspond to the duodenum, jejunum and ileum, respectively. The portions were cut transversely into approximately 3 mm pieces, and post-fixed in the same fixative for 2 h at room temperature. Subsequently, the tissue was washed with PBS and incubate in a solution of 20 mM DTT, 20% ethanol, and 150 mM Tris pH 8.0, for 1 h at room temperature to clear it of mucus. The pieces of intestinal tissue were then incubated in a solution of 30% sucrose in PBS overnight at 4°C, embedded in Andwin Scientific Tissue-Tek CRYO-OCT Compound (Fisher Scientific, Pittsburg, PA), and frozen at −70°C until further use. Transverse sections (20 μm) were obtained with a cryostat and collected in PBS. The sections were incubated first in blocking solution (0.1% Triton X-100, 1% heat-inactivated goat serum in PBS) for 1 h, and then with a particular primary antibody diluted in blocking solution, overnight at 4°C. Primary antibodies were as follows: 1:500 dilution of rat anti-PLP/DM20 (gift from Wendy Macklin); 1:1000 dilution of rabbit anti-GFAP (Novus Biologicals, Centennial, CO, Cat# NB300-141). After which, the free-floating sections were washed three times in PBS (30 min each) and incubated with a species-appropriate secondary antibody for 1 h at room temperature: 1:1000 dilution of goat anti-rat Alexa-Fluor 594 (ThermoFisher Scientific/Invitrogen, Cat# A11007); 1:1000 dilution of goat anti-rabbit Alexa-Fluor 488 (Abcam, Cat# ab150077). The sections were washed again, three times in PBS, and mounted in VECTASHIELD Antifade Mounting Medium with DAPI (Vector Laboratories, Burlingame, CA, Cat# H-1200-10). Images were captured on a Zeiss LSM 880 confocal microscope with ZEN blue 3.2 software (Carl Zeiss Microscopy, White Plains, NY).

Whole mount sections of intestine were obtained using a protocol adapted from Ahrends et al. (2022). Briefly, the entire gastrointestinal tract beginning at the lower esophagus through the rectum was removed from a euthanized mouse at P21 of age and rinsed in ice cold PBS. Next, regions of the gastrointestinal tract (duodenum, jejunum, ileum and colon) were separated, opened, and pinned flat with the luminal side up on a Sylgard dissection dish (Living Systems Instrumentation, St. Albans, VT, Cat# DD-90-S-BLK). Under a dissecting microscope, fine forceps were used to dissect the layers of intestine. The peeled tissue sections were incubated in fixative (4% paraformaldehyde in PBS) for 16 h at 4°C, washed four times in PBS (10 min each), and then incubated in a solution of 20 mM DTT, 20% ethanol, and 150 mM Tris pH 8.0, overnight at 4°C to clear the tissue of mucus. Subsequently, the floating sections were transferred to Permeabilization Buffer (0.2% Triton-X 100 in PBS) for 2 h at room temperature on a shaker, washed three times in PBS (10 min each), and incubated in blocking buffer [5% bovine serum albumin, 5% normal goat serum (Jackson ImmunoResearch, West Grove, PA, Cat# 005-000-121), 0.2% Triton-X 100 in PBS] for 1 h at room temperature. The free-floating sections were then incubated with a particular primary antibody for 48 h at 4°C and processed further as described above for transverse sections.

Reference

Ahrends, T., Weiner, M., and Mucida, D. (2022). Isolation of myenteric and submucosal plexus from mouse gastrointestinal tract and subsequent flow cytometry and immunofluorescence. *STAR Protocols* 3, 101157. doi: 10.1016/j.xpro.2022.101157
