# Supplementary figures and images for: Plp1 in the enteric nervous system is preferentially expressed during early postnatal development in mouse as DM20, whose expression appears reliant on an intronic enhancer
Source: Front Cell Neurosci. 2023 May 24;17:1175614. doi: 10.3389/fncel.2023.1175614 (PMC10244531; doi:10.3389/fncel.2023.1175614)

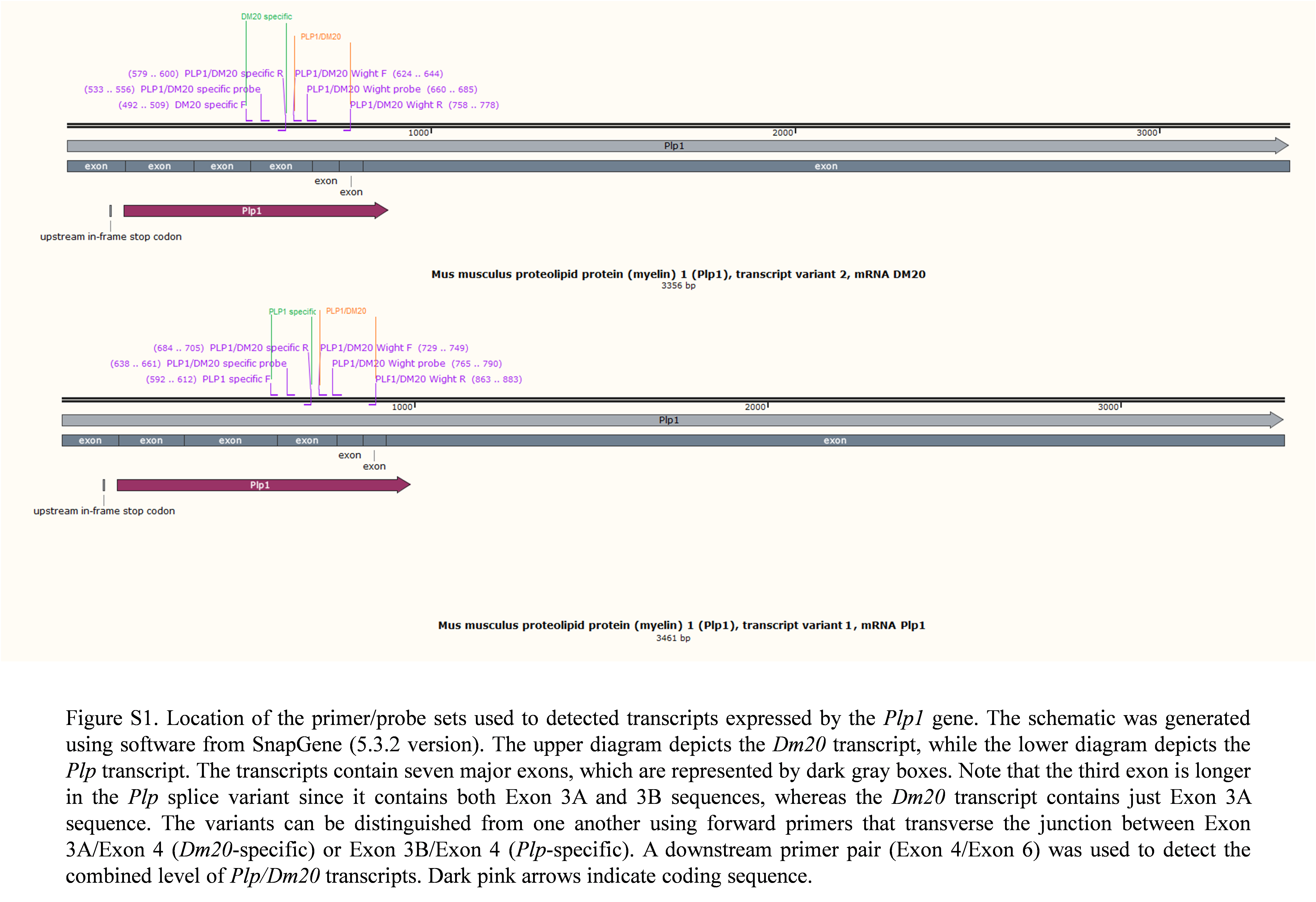

Supplement: Supplementary file 2 [file Image_1.TIF]

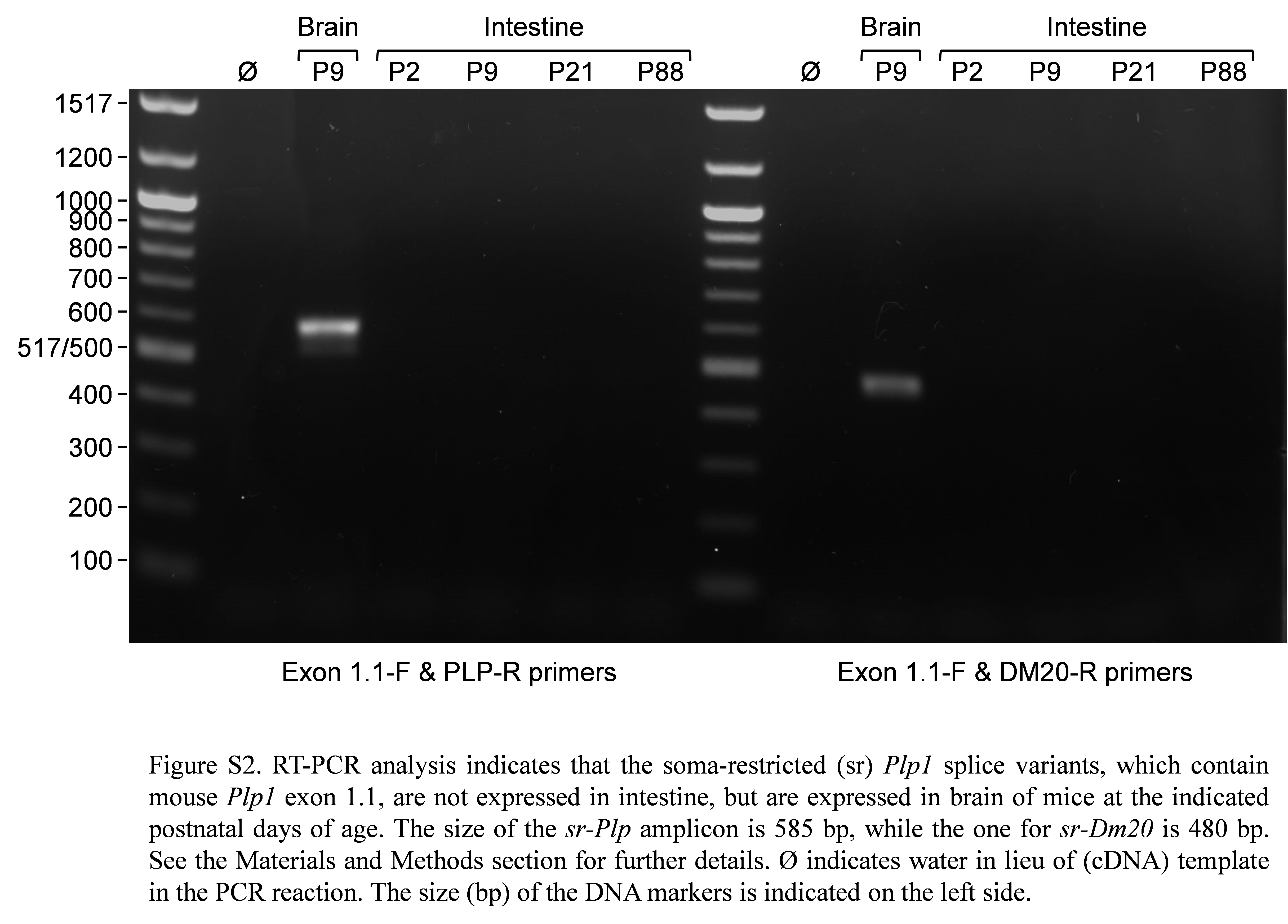

Supplement: Supplementary file 3 [file Image_2.TIF]

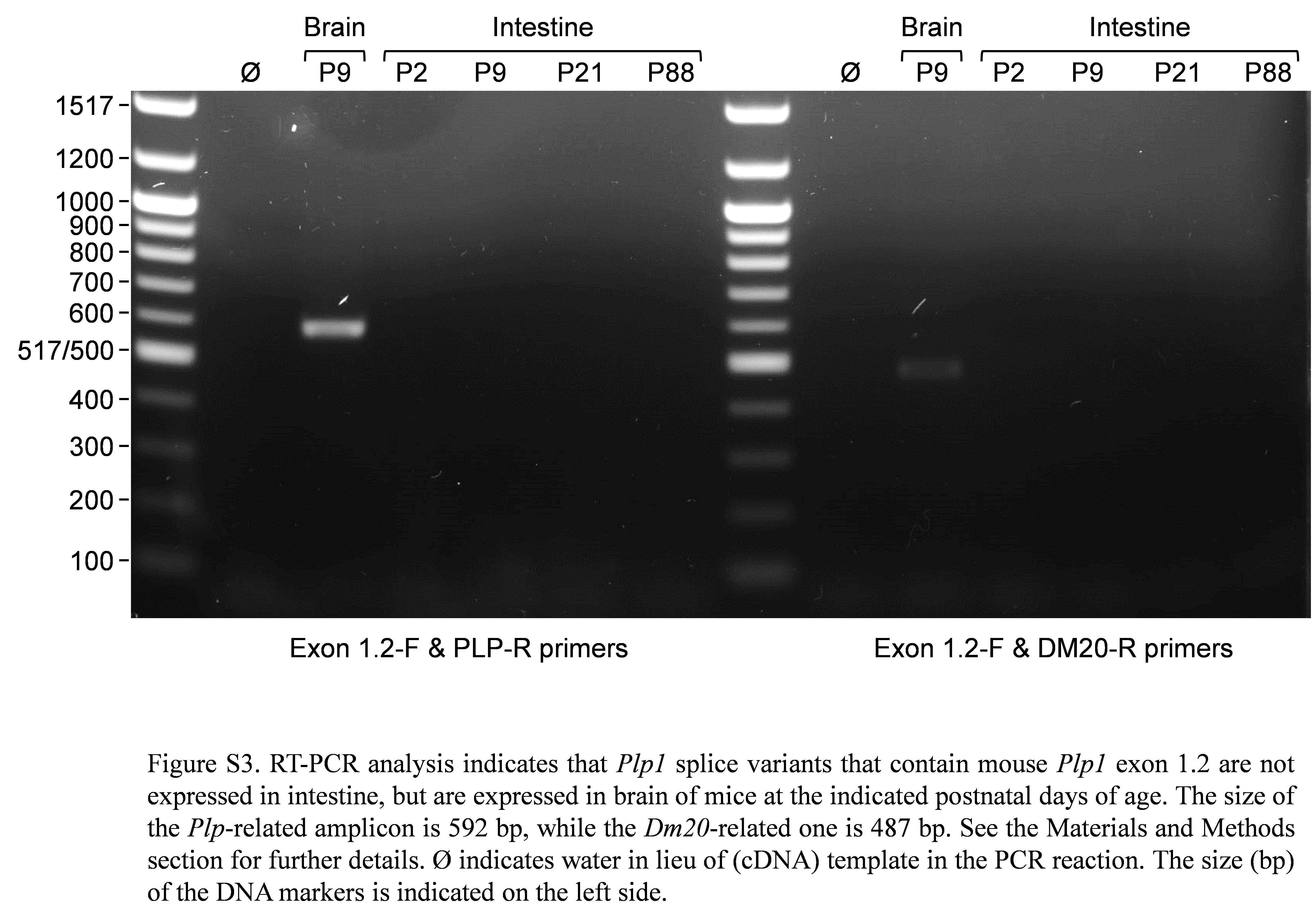

Supplement: Supplementary file 4 [file Image_3.TIF]

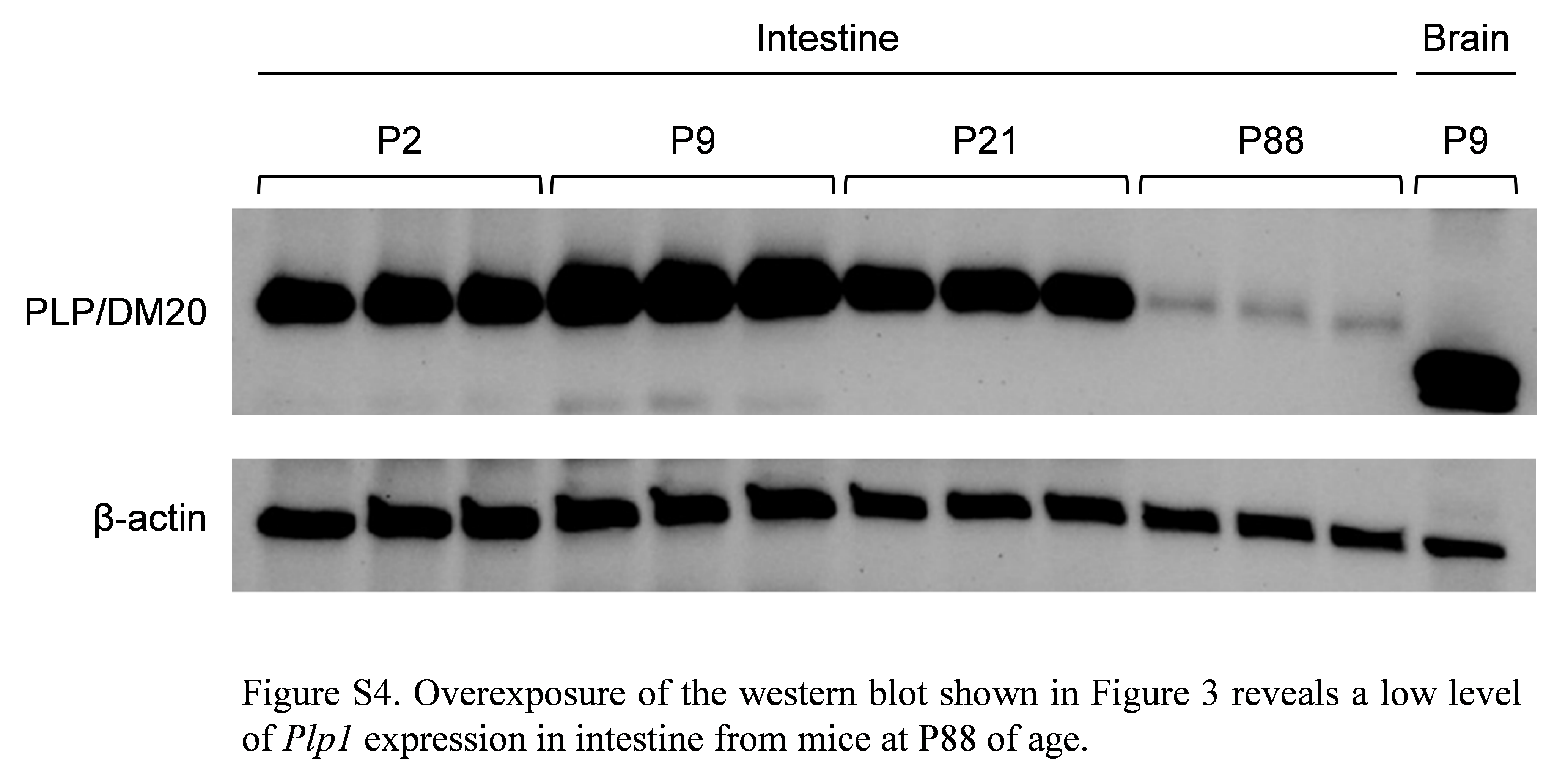

Supplement: Supplementary file 5 [file Image_4.TIF]

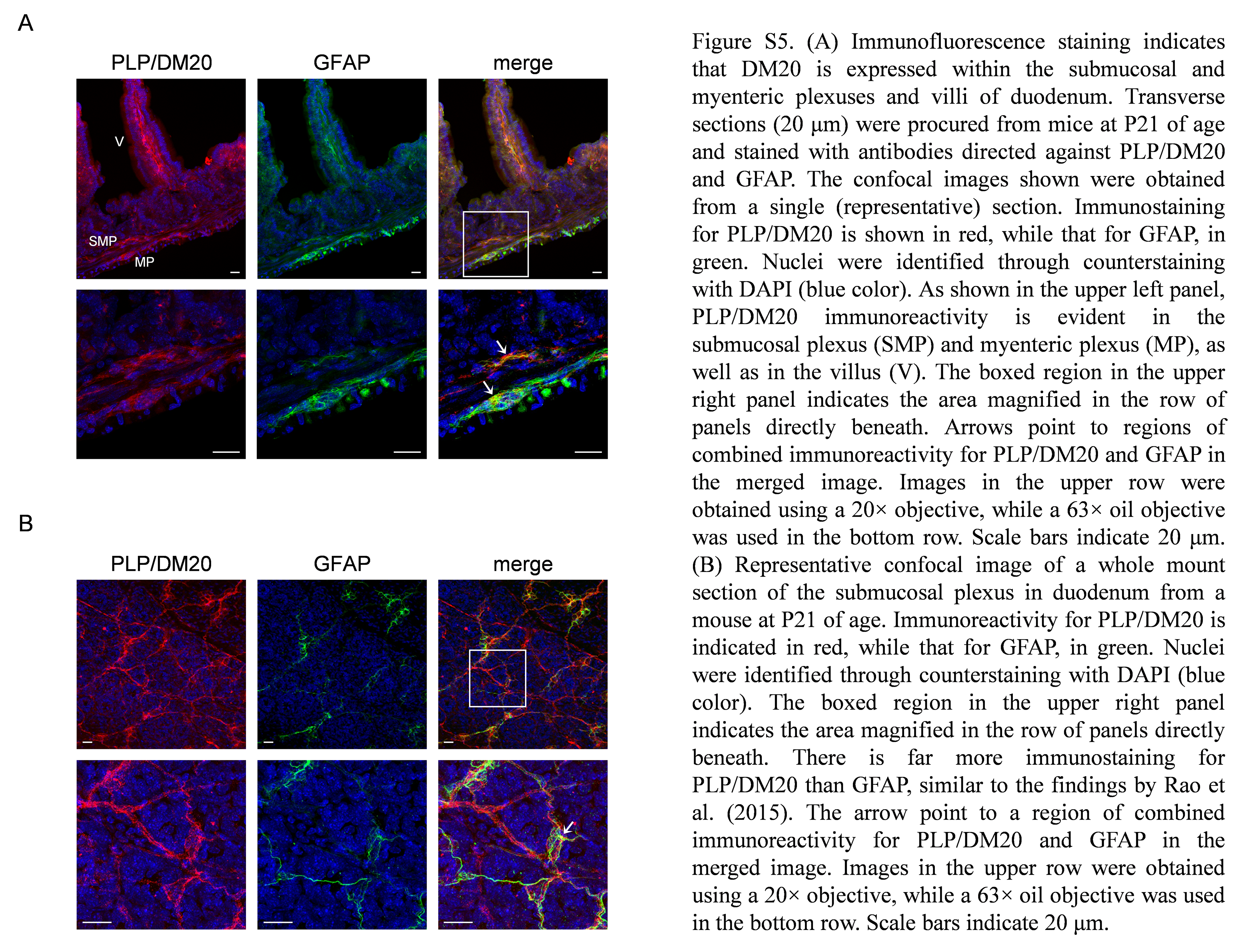

Supplement: Supplementary file 6 [file Image_5.TIF]
